# Oldest Known *Eucalyptus* Macrofossils Are from South America

**DOI:** 10.1371/journal.pone.0021084

**Published:** 2011-06-28

**Authors:** María A. Gandolfo, Elizabeth J. Hermsen, María C. Zamaloa, Kevin C. Nixon, Cynthia C. González, Peter Wilf, N. Rubén Cúneo, Kirk R. Johnson

**Affiliations:** 1 L.H. Bailey Hortorium, Department of Plant Biology, Cornell University, Ithaca, New York, United States of America; 2 Departamento de Ecología, Genética y Evolución, Universidad de Buenos Aires, Capital Federal, Buenos Aires, Argentina; 3 Museo Paleontológico Egidio Feruglio-Consejo Nacional de Investigaciones Científicas y Técnicas, Trelew, Chubut, Argentina; 4 Department of Geosciences, Pennsylvania State University, University Park, Pennsylvania, United States of America; 5 Denver Museum of Nature and Science, Denver, Colorado, United States of America; Raymond M. Alf Museum of Paleontology, United States of America

## Abstract

The evolutionary history of *Eucalyptus* and the eucalypts, the larger clade of seven genera including *Eucalyptus* that today have a natural distribution almost exclusively in Australasia, is poorly documented from the fossil record. Little physical evidence exists bearing on the ancient geographical distributions or morphologies of plants within the clade. Herein, we introduce fossil material of *Eucalyptus* from the early Eocene (ca. 51.9 Ma) Laguna del Hunco paleoflora of Chubut Province, Argentina; specimens include multiple leaves, infructescences, and dispersed capsules, several flower buds, and a single flower. Morphological similarities that relate the fossils to extant eucalypts include leaf shape, venation, and epidermal oil glands; infructescence structure; valvate capsulate fruits; and operculate flower buds. The presence of a staminophore scar on the fruits links them to *Eucalyptus*, and the presence of a transverse scar on the flower buds indicates a relationship to *Eucalyptus* subgenus *Symphyomyrtus*. Phylogenetic analyses of morphological data alone and combined with aligned sequence data from a prior study including 16 extant eucalypts, one outgroup, and a terminal representing the fossils indicate that the fossils are nested within *Eucalyptus*. These are the only illustrated *Eucalyptus* fossils that are definitively Eocene in age, and the only conclusively identified extant or fossil eucalypts naturally occurring outside of Australasia and adjacent Mindanao. Thus, these fossils indicate that the evolution of the eucalypt group is not constrained to a single region. Moreover, they strengthen the taxonomic connections between the Laguna del Hunco paleoflora and extant subtropical and tropical Australasia, one of the three major ecologic-geographic elements of the Laguna del Hunco paleoflora. The age and affinities of the fossils also indicate that *Eucalyptus* subgenus *Symphyomyrtus* is older than previously supposed. Paleoecological data indicate that the Patagonian *Eucalyptus* dominated volcanically disturbed areas adjacent to standing rainforest surrounding an Eocene caldera lake.

## Introduction

**Figure 1:**
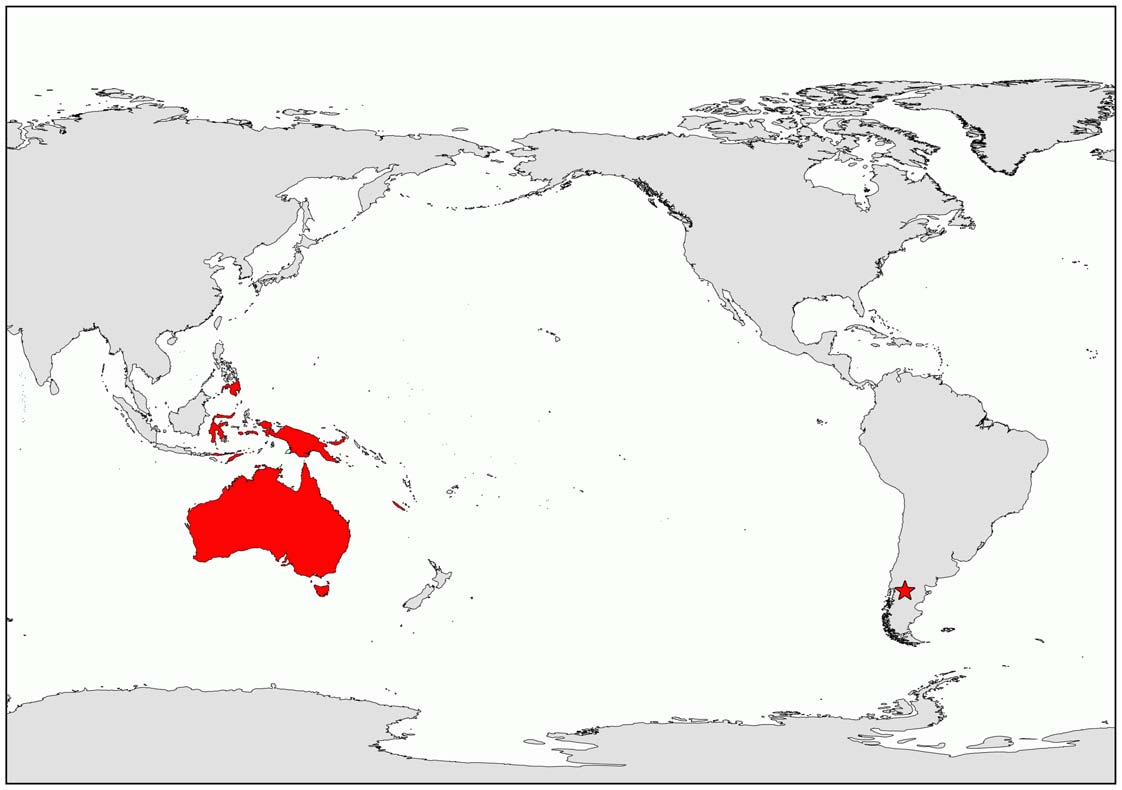
Distribution of extant eucalypts after Ladiges et al. [**7**] (shaded regions in Australasia and adjacent island of Mindanao) and present position of the early Eocene Laguna del Hunco localities, Chubut Province, Argentina (star).

The early Eocene (ca. 51.9 Ma) Laguna del Hunco biota of the Tufolitas Laguna del Hunco, northwestern Chubut Province, Patagonia, Argentina (Fig. 1), is considered one of the most biodiverse Cenozoic fossil deposits worldwide. The biota is composed of extraordinarily rich assemblages of plant, insect, and vertebrate fossils representing organisms that flourished in an ancient caldera lake system [1]–[6]. Among the thousands of specimens recovered from Laguna del Hunco [2], we investigated a suite of fossils that exhibit characters of the living genus *Eucalyptus* L'Hérit. This genus, including over 600 species of trees and shrubs of the flowering plant family Myrtaceae Juss. (Myrtle Family) [7], is an iconic and dominant component of the Australian vegetation; it is arguably the most important genus in Australia both in standing biomass and number of species, and it has a very broad ecological range from desert to tropical rainforest margins [8]. *Eucalyptus* is part of a monophyletic group of seven genera, hereafter informally referred to as eucalypts, that are endemic to Australasia; only one of the species ranges north beyond this region to the Philippine island of Mindanao [7]. The clade includes the monotypic genera *Allosyncarpia* S.T. Blake, *Arillastrum* Pancher ex. Baill., and *Stockwellia* D.J. Carr, S.G.M. Carr & B. Hyland; the small genera *Eucalyptopsis* C. White (2 species) and *Angophora* Cav. (13 species); and the largest genera, *Corymbia* K.D. Hill & L.A.S. Johnson (113 species) and *Eucalyptus* (>600 species) [7], [9]. Despite their present diversity and ecological and economic importance, the origin and early evolution of *Eucalyptus* and the other eucalypts is not well understood, in part because the eucalypt fossil record consists primarily of reports that are incomplete or have poor temporal constraints.

Morphologically, eucalypts are characterized by a unique umbel-like inflorescence structure, the umbellaster, which always bears an odd number of flowers; woody capsulate fruits that open by valves; and curved, slightly to dramatically asymmetrical leaves [10]–[14]. Traits of the bark, flowers, stamens, fruits, and leaves are commonly used for distinguishing eucalypt genera and species. Distinctive leaf characters include venation pattern, presence or absence of an intramarginal vein, distribution of oil glands, and color [10], [12], [13]. Floral characters include presence or absence of a pedicel, number of perianth whorls, presence of calycine or corolline opercula (floral caps formed by the sepals and/or petals), and retention or loss of one or both perianth whorls or of the distal portion of the hypanthium before, at, or following anthesis [10]–[12], [15]. The fruit is the single most useful structure for differentiating species, particularly within *Eucalyptus*; important characters include size and shape, the orientation of the disk, and the disposition and number of valves [10]–[14].

## Results

The fossils reported herein are impressions of flower buds, a flower, individual fruits, infructescences, and leaves. Although the different organ types are not in organic connection, they were found repeatedly associated at the same stratigraphic levels and quarry sites, and on single slabs. Given their co-occurrence, we infer that many of these organs were produced by the same plant taxon.

**Figure 2:**
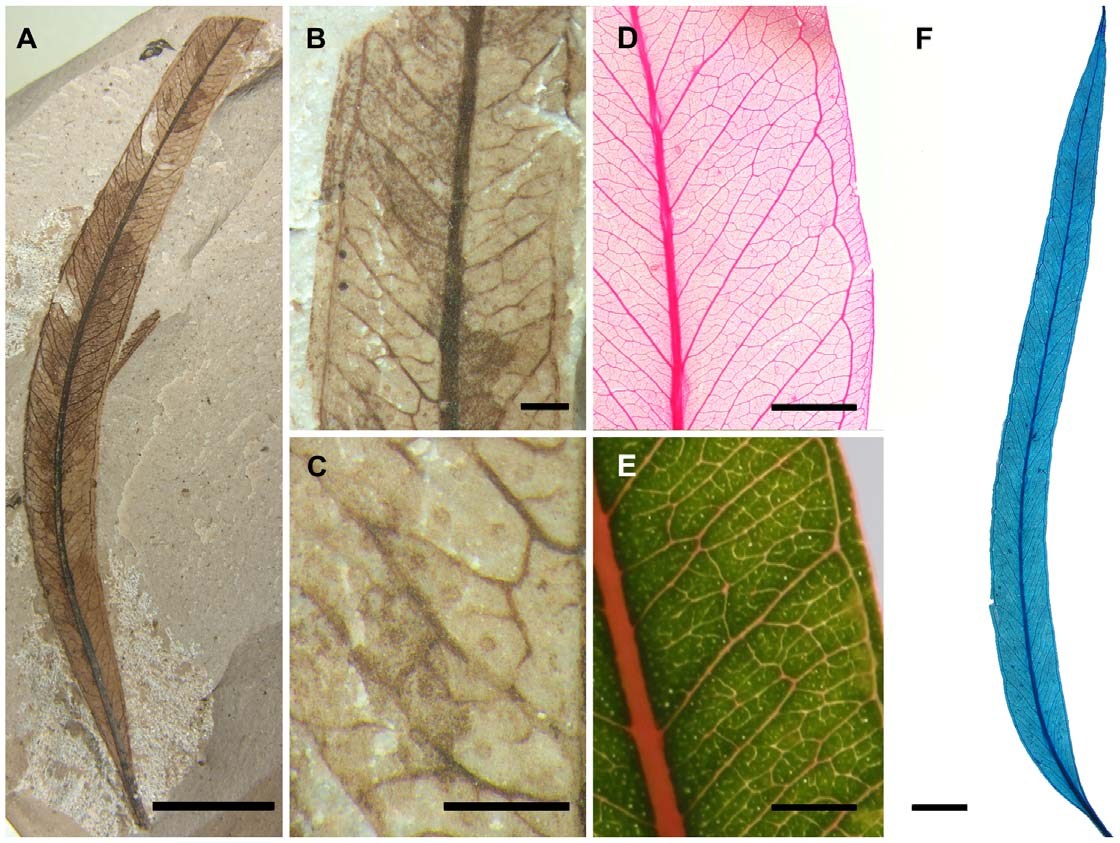
*Eucalyptus* leaves. A–B. MPEF-Pb 2329, A. Overall view showing the linear to lanceolate shape, the acute and acuminate apex, and the acute and decurrent base. B. Detail of the venation pattern; note the intramarginal, secondary, and intersecondary veins. C. MPEF-Pb 3729, detail of lamina showing island oil glands and higher order venation pattern. D. *E. bridgesiana* R.T. Baker, BH 37791, detail of the higher order venation pattern. E. *E. camaldulensis* Dehnh, detail of the lamina showing the island oil glands. F. *E. tereticornis* Sm., PBP-1003, overall view of a cleared leaf; note the general morphology of the lamina and the venation pattern. Scale bars: A, F, 1 cm; B–C, 1 mm; D–E, 5 mm.

The fossil leaves are arranged alternately on branches, and each has a robust marginal petiole. The leaves are simple microphylls to notophylls, 4–10.5 cm long and 0.4–1.9 cm wide (length/width: 4.5–10), linear to lanceolate in shape, always falcate, and slightly to strongly asymmetrical. The leaf apex is acute and acuminate, while the base is acute and decurrent (Fig. 2A). The margin is entire. The lamina shows numerous minute dark dots scattered within the areoles that are interpreted as oil glands; because these glands are not associated with a veinlet, they can be classified as isolated or island glands (Figs. 2B, C). The primary venation is pinnate, and the midvein is simple and straight. There are no agrophic veins. The secondary venation is composed of 15 to 30 pairs of craspedodromous secondary veins; the secondary veins are subparallel, regularly spaced, and emerge smoothly from the midrib at acute angles that increase from the base towards the apex (40°–45° at the base and 70°–75° at the apex). They terminate in a strong intramarginal vein that closely parallels the margin. Intersecondary veins are sometimes present paralleling the secondary veins; each is less than 50% as long as its subjacent secondary vein, and never bifurcates (Figs. 2A, B). The higher order venation is reticulate; the tertiary veins are admedially ramified, forming a well-defined net; the quaternary venation is freely ramifying. The areoles are poorly to moderately developed and 3-, 4-, or 5-sided (Figs. 2B, C). The marginal ultimate venation is looped, forming a single line of areoles between the margin and the intramarginal vein (Figs. 2A, B).

**Figure 3 pone-0021084-g003:**
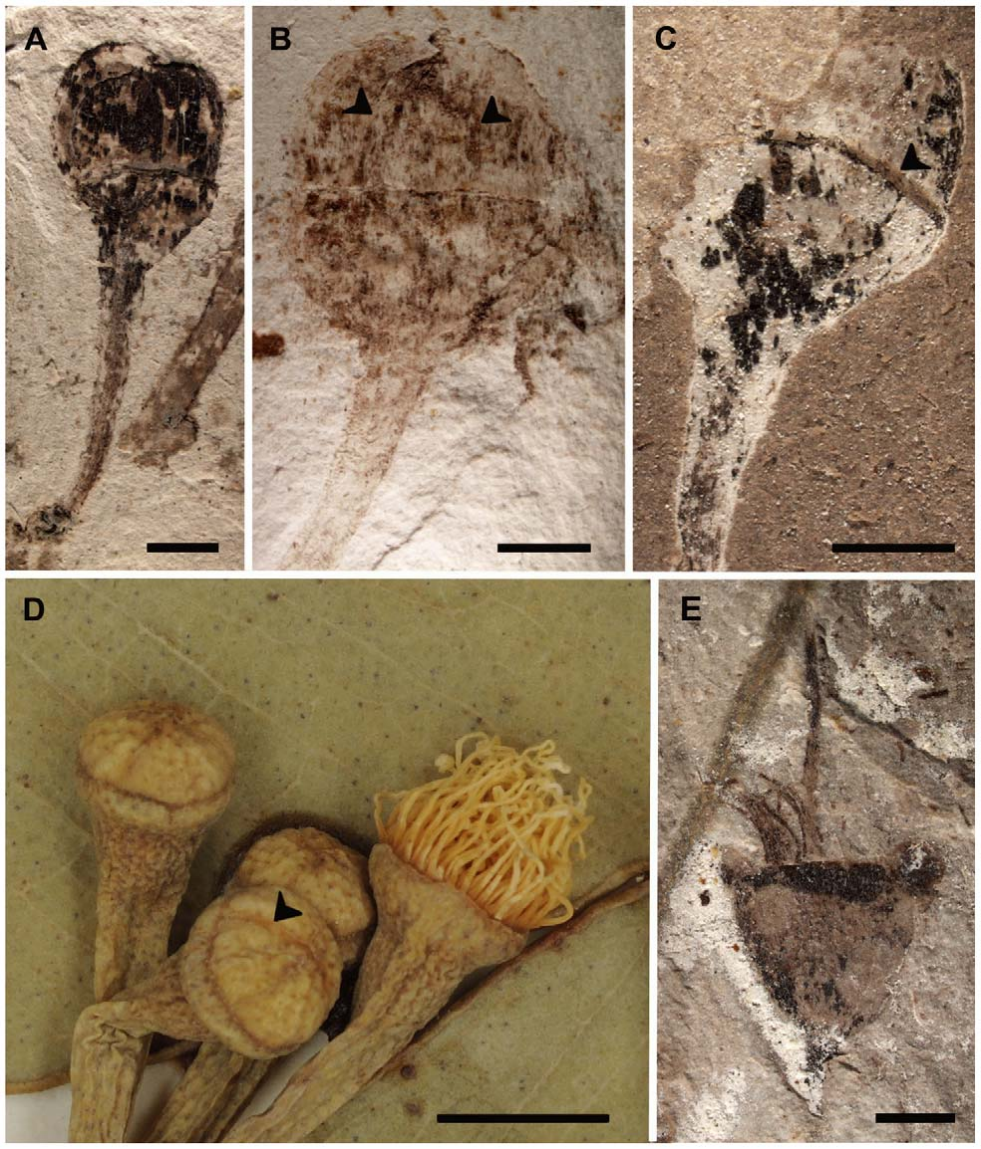
*Eucalyptus* flower buds and flower. A. MPEF-Pb 3735, overall view of flower bud showing corolline operculum and transverse scar left after loss of the calyx. B. MPEF-Pb 3733, flower bud with hemispherical hypanthium and corolline operculum of coherent petals (arrows show petal margins). C. MPEF-Pb 3734, calycine scar (arrow) denotes the separation between the hypanthium and corolline operculum. D. *E. microcorys*, BH 37596, flower buds and open flower; note the hypanthium, and corolline operculum of coherent petals with visible margins (arrow) on the flower buds. E. MPEF-Pb 3738, bisexual flower with single style and stigma and numerous stamens. Scale bars: A–C, E, 2 mm; D, 3 mm.

Fossil flower buds are globose and pedicellate, 0.5–0.8 cm in height and a maximum of 0.6 cm in width. The hypanthium varies in shape, lacks ribs, is apparently smooth, and measures approximately 0.3 cm in height and 0.4–0.6 cm in width; it is capped by a corolline operculum (Figs. 3A–C)—the inner or sole floral cap formed by the coherent or connate petals in *Corymbia* and *Eucalyptus* [9], [11], [16], [17]—that is 0.3–0.5 cm in height. The flowers are inferred to have produced a calyx, based on the presence of a calycine scar marking the separation between the hypanthium and corolline operculum (Figs. 3A–C). This type of scar is a hallmark of eucalypt flowers that initially have two perianth whorls but lose the outer, calycine whorl early in development (Fig. 3D) [10]–[12]. The open flower is bisexual and lacks sepals and petals (Fig. 3E), suggesting that the corolline operculum is shed as in all extant species of *Corymbia* and *Eucalyptus* (Fig. 3D) [11]; the inferior ovary is sunken within the hypanthium. There is a single style that terminates in a simple stigma, and numerous stamens probably surrounded the ovary (Fig. 3E).

**Figure 4:**
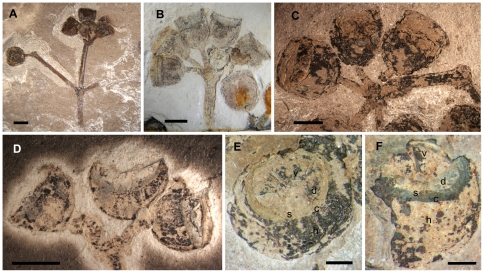
*Eucalyptus* fossil infructescences. A. MPEF-Pb 3750, infructescence composed of two umbellasters. B. MPEF-Pb 981, capsules in unbellaster. C. MPEF-Pb 3740, infructescence showing the morphology and orientation of the fruit disks. D. MPEF-Pb 3739, infructescence composed of at least three fruits showing valves and disks. E–F. MPEF-Pb 2374. E. Top view of a capsule showing corolline operculum scar (c), staminophore scar (s), valves (v), disk (d), and hypanthium (h). F. Side view of capsule showing the same features. Scale bars: A, B, D, 3 mm; C, 2 mm; E–F, 1 mm.

Fossil fruits are pedicellate and clustered in umbellasters (umbel-like inflorescence structures), which are sometimes grouped together in compound fertile branches (Fig. 4A). Each umbellaster bears three, five, or seven woody capsules (Figs. 4A–D). Each capsule has a rim interpreted as the corolline operculum scar, a thinner staminophore (a distinct, raised tissue that bears the stamens) scar, and a broad disk (Figs. 4C–F). The valves are retained on the apex of the ovary when the capsule opens and vary in number from five to six; they may or may not protrude above the rim of the capsule (Figs. 4E–F). When the capsule is preserved in partial apical view, the edges of the valves are visible (Fig. 4E); the disk appears to be at or rising above rim level in some specimens (Figs. 4C, D, F).

**Figure 5:**
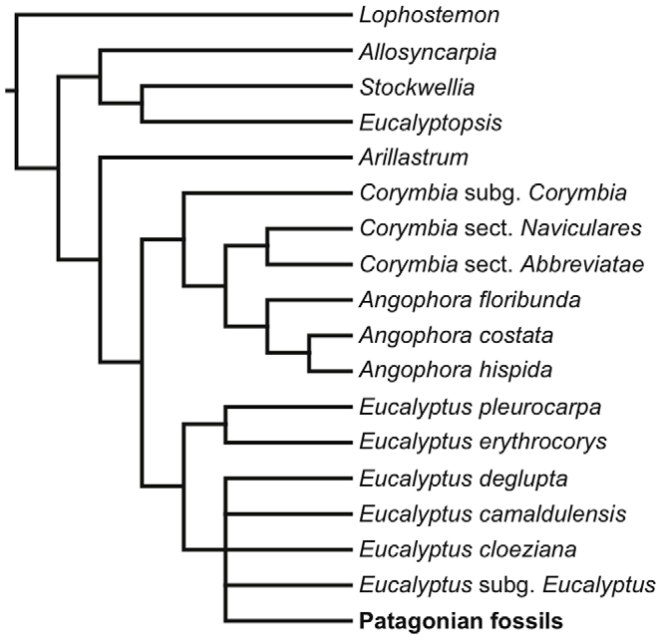
Strict consensus tree. Strict consensus tree of five most parsimonious trees (length 381steps, CI 0.66, RI 0.78) found during simultaneous analysis of morphological and molecular sequence data. Terminal names for *Corymbia* subgenera and sections are after Parra-O. et al. [9]; the subgeneric terminal name for *Eucalyptus* is after Brooker [66]. For a full list of exemplar taxa used in the analysis, see Table S1.

Results of a combined molecular and morphological phylogenetic analysis of extant eucalypts with a terminal representing the characteristics of the fossils differ somewhat from other recent studies [9], [18] by indicating that *Corymbia* is paraphyletic with respect to *Angophora* rather than a separate, monophyletic genus. We note, however, that monophyletic *Corymbia* is not universally supported in analyses of molecular sequence data [19]–[21]. The strict consensus tree from our analysis indicates that the fossils are nested within the clade that includes all extant members of the genus *Eucalyptus*, although the position of the terminal representing the Patagonian fossils is somewhat unresolved (Fig. 5). Results of an analysis of morphological characters alone are similar in suggesting that the fossils belong within the crown-group *Eucalyptus* clade, although in a somewhat unresolved position (Fig. S1). Thus, we interpret the fossils as belonging within the clade including extant *Eucalyptus* species. Further, the fossils are almost certainly allied to *Eucalyptus* subgenus *Symphyomyrtus* (Schauer) Brooker, the largest of the *Eucalyptus* subgenera, because the fossil flower buds lack sepals and show a calyx scar. Within the *Eucalyptus* clade, the presence of a calyx scar is a character exclusive to ingroup *Symphyomyrtus* and one closely related species, *E. guilfoylei* Maiden [10]–[12], [16], [22]. In some *Eucalyptus* with free caducous sepals, there is no calycine scar (Fig. 3D); these taxa include *E. cloeziana* F. Muell. (monotypic subgenus *Idiogenes* L.D. Pryor & L.A.S. Johnson ex Brooker), *E. microcorys* F. Muell. (subgenus *Alveolata* (Maiden) Brooker, but thought to be a stem symphyomyrt [20]), and *E. tenuipes* (Maiden & Blakely) Blakely & C.T. White (subgenus *Cuboidea* Brooker, whose relationships are poorly understood [20]) [12], [23]. In other *Eucalyptus*, the flower bud is completely smooth, or the sepals are persistent [10]–[12].

## Discussion

The morphological characters exhibited by these fossils leave little doubt that they belong within the eucalypts. The characters preserved in the fossil leaves are consistent with those present in extant eucalypts (Figs. 2E–F), while the features of the flowers and fruits are consistent with those of members of the *Angophora-Corymbia-Eucalyptus* subclade. Among eucalypts, the flower bud lacking sepals and displaying a transverse calyx scar is characteristic of *Corymbia* subgenus *Blakella* (L.D. Pryor & L.A.S. Johnson ex Brooker) Parra-O. & Ladiges, *E. guilfolyei* (probable stem-group *Symphyomyrtus* [20], [24]), and much of the large subgenus *Symphyomyrtus* (ca. 450 species) of *Eucalyptus* [9]–[12], [16], [17], [22]. The fossil fruits are inconsistent with fruits of *Corymbia*; the presence of a staminophore is a characteristic restricted to *Eucalyptus* and lacking in *Corymbia* [25]. Among other differences, *Corymbia* fruits generally have a disc that slopes downward and valves that are below the level of the capsule rim [10], [12], [17]. *Eucalyptus* fruits may have a disc that slopes downward, upward, or is level with the capsule rim, and valves that vary from below rim level to protruding [10], [12].

In most species of *Eucalyptus* with corolline opercula, the operculum forms very early in development, and the edges of the individual petals are obscure in the fully developed flower bud (although in some species fusion is delayed and individual petal apices can be discerned) [16]. In contrast, the petal edges are obvious in the Patagonian fossil flower buds (Fig. 3B). Among *Eucalyptus*, only five species are documented to have corolline opercula with externally discernible, weakly fused, imbricate petals (*E. brachyandra* F. Muell., *E. curtisii* Blakely & White, *E. guilfolyei*, *E. microcorys*, and *E. tenuipes* [12], [16], [22], [26]), and at least three of these (*E. brachyandra*, *E. guilfolyei*, and *E. microcorys*) are within or thought to be near subgenus *Symphyomyrtus* [20], [21], [24], [27]. In these extant taxa, the cuticles of the petals composing the corolline operculum cohere, but the petals do not truly become united into a single structure during development (e.g., the petals are coherent but not connate) (Fig. 3D) [16], [26], perhaps suggesting a parallel condition in the fossils. Notably, in one fossil flower bud where three petals are clearly visible, the central petal appears to be markedly shorter than the lateral petals (Fig. 3B), likely because it is partially obscured due to imbrication of the petals. A similar condition can be observed in flower buds of extant species with coherent petals such as *E. guilfoylei* and *E. microcorys* [16], [22]. No extant species that has coherent petals is also known to possess a staminophore [16], [26] as in the fossils.

Temporal estimates based on a vicariance model of extant eucalypt distribution (whereby the New Caledonian endemic *Arillastrum* lineage is inferred to have diverged from the remainder of the clade due to rifting between Australia and the block containing New Caledonia beginning in the Late Cretaceous) as well as evidence of eucalypt-type pollen in the early Paleogene of Australia have been used to suggest that the eucalypts probably arose by the Late Cretaceous (65–85 Ma) [7], [28]. A more recent reevaluation of the geologic history of the region suggests, however, that the biotas of New Caledonia and Australia may have maintained a land-based connection into the Cenozoic [29]. The known macrofossil record of the eucalypts does not begin until the Cenozoic and is relatively sparse even in Australia, despite the dominance of the eucalypts in the flora today [30]. The oldest definitive evidence of eucalypt macrofossils (as judged by a combination of sufficient characters and good temporal constraints) is considered by some to be either an early Miocene petrified stump from New South Wales [30] or late Oligocene leaves that are reported to co-occur with undescribed capsules from Victoria [31]. The fossil record of reproductive material is not well documented. The oldest and only Australasian occurrences of eucalypt reproductive structures with narrow age constraints that are fully described and figured are from the early to middle Miocene of Australia and New Zealand [32], [33]. Possibly the oldest reproductive macrofossil evidence of the eucalypts, however, is an infructescence bearing capsules similar to those of *Angophora* and *Corymbia* from the Redbank Plains Formation of Queensland, which may be as old as Paleocene in age [34]. This specimen has not been analyzed in detail. Additional eucalypt reproductive material reportedly occurs in early to middle Eocene sediments of Australia, although these fossils are currently unfigured and undescribed [35], [36] or are imprecisely dated, possibly being as young as Neogene in age [37]–[39]. The Patagonian fossils described herein thus represent the oldest suite of eucalypt macrofossils that includes figured reproductive material and has a well-constrained age: ^40^Ar-^39^Ar analyses on sanidine from a tuff bed stratigraphically intercalated with the Laguna del Hunco fossil quarries yielded an age of 51.91±0.22 Ma (early Eocene), and this is corroborated by two other ^40^Ar-^39^Ar dates and paleomagnetic data [1], [2].

Because the age of these fossils is well-constrained and because the fossils have been placed in phylogenetic context, they can be used to test the results of recent molecular dating studies in which the age of the eucalypts or eucalypt subgroups has been calculated. The age of the fossils refutes a more recent 35–45 Ma estimate for the age of crown-group eucalypts that was calculated using the ages of multiple fossil occurrences as calibration points in a molecular phylogeny of Myrtaceae, including a 48 Ma calibration point based on the occurrence of the aforementioned Redbank Plains Formation infructescence [40]. These fossils also predate an estimated 41–46 Ma age range for the origination of stem-group *Symphyomyrtus*, based on a molecular phylogenetic dating study employing the assumption that crown-group eucalypts are Late Cretaceous in age [28]; however, in order to fully refute or corroborate the stem-group *Symphyomyrtus* age range, the fossils must be placed in more precise phylogenetic context with relation to the subgenus *Symphyomyrtus*.

In addition to their importance for understanding the morphological evolution of *Eucalyptus* and the age of the eucalypts, these fossils provide valuable biogeographic and paleoenvironmental information. Biogeographically, most species in the eucalypt clade have distributions confined to Australia; exceptions, in addition to *Arillastrum*, include both species of *Eucalyptopsis*, from Indonesia and Papua New Guinea; two species of *Corymbia* found in Papua New Guinea; and five species of *Eucalyptus* subgenus *Symphyomyrtus* distributed in Indonesia, the Philippines (Mindanao), and Papua New Guinea [7], [17] (Fig. 1). The fossil record of the eucalypts was previously known to extend their ancient distribution to New Zealand [33], with one additional specimen providing tenuous support for a past distribution in South America [41]. The previously reported South American *Eucalyptus*, *E. patagonicus* Frenguelli, is based on a single, poorly preserved specimen from Neuquén Province, Argentina; it consists of an umbellaster with three mature globose fruits. Although the fossil shows similarities to the eucalypts, its placement is much less certain than that of the fossils described in this paper. Johnson & Briggs [42] wrote that the specimen “could conceivably” have affinities to subgenus *Symphyomyrtus* based on illustrations of it, but they did not detail the characters that might link it to that subgenus. The provenance and age of the Neuquén material is also ambiguous; originally described as Miocene [41], it may be as old as Eocene in age [30]. Other Patagonian fossils thought to potentially represent *Eucalyptus* or the eucalypts—including leaves assigned to *Myrcia chubutensis* Berry [41], [43], fruits, and a possible flower bud—have been rejected [42]. Thus, the *Eucalyptus* fossils reported here are currently the only reliable record of any eucalypt occurring naturally outside of Australasia and adjacent Mindanao.

The biogeographic pattern where genera are found in the Eocene of Patagonia that today inhabit Australasia is exemplified by *Eucalyptus* and other Laguna del Hunco taxa, including the extant angiosperm genera *Gymnostoma* L. Johnson (Casuarinaceae R. Br.) and *Akania* Hook. f. (Akaniaceae Stapf.) and the extant conifer genus *Papuacedrus* Li (Cupressaceae Gray) [44]–[46]. Due to the relatively derived position of the fossils within the genus *Eucalyptus*, they do not provide evidence that *Eucalyptus* originated outside of Australasia, despite their antiquity. The presence of *Eucalyptus* in Eocene South America, however, adds a new dimension to what was once a regionally limited understanding of the biogeographic history of the genus and suggests that *Eucalyptus* also once occurred on Antarctica, because this continent served as a connection between Australia and South America during the Paleogene [47].

Interbedded volcanics and the high tuff content of the strata preserving the Laguna del Hunco biota indicate that Laguna del Hunco was a dynamic caldera system that was subject to frequent disturbance from lava flows, earthquakes, and landslides [1], [48] that would have destroyed closed rainforest. Because extant *Eucalyptus* is characterized by shade-intolerant seedlings requiring open, disturbed habitats [8], the abundant (see Materials & Methods) *Eucalyptus* fossils at Laguna del Hunco suggest a vegetational mosaic wherein *Eucalyptus* colonized and dominated disturbed areas alongside intact rainforest. A possible analog is living species of subgenus *Symphyomyrtus* from island arcs north of Australia that proliferate on recent lava flows and older volcanic soils adjacent to standing rainforest, such as *E. deglupta* Blume and *E. urophylla* S.T. Blake [8], [49], [50]. Since these living species are apparently not closely related to each other [20], [21], this ecological strategy has probably arisen more than once during the evolutionary history of the eucalypts. The latter hypothesis is further supported by the existence of Miocene eucalypt fossils from New South Wales, Australia, which are also preserved in lacustrine sediments interbedded with tuffaceous strata [32].

## Materials and Methods

### Specimen and Repository Information

*Eucalyptus* fossils collected from Tufolitas Laguna del Hunco localities LH4, LH6, LH13, LH15, LH25, and LHF (denoting specimens found in float) [1], as well as new locality LH27 (at the same stratigraphic level as LH6), are housed in the Paleobotanical collection of the Museo Paleontológico Egidio Feruglio (MPEF), Trelew, Chubut Province, Argentina, under these numbers: leaves, MPEF-Pb 3726, 3729–3732; flower buds, MPEF-Pb 3733–3737; flower, MPEF-Pb 3738; and infructescences, MPEF-Pb 3727–3728, 3739–3759. Abundant additional material of *Eucalyptus* leaves is held at MPEF but is not formally assigned here; most corresponds to morphotype TY21 of Wilf et al. [2], who tabulated that it comprised 534 of 4,303 (10.1%) field-censused plant fossil specimens from Laguna del Hunco. Other material not assigned specific locality information but collected from the Tufolitas Laguna del Hunco is housed in the paleobotanical collection of the Departamento de Ecología, Genética y Evolución, Facultad de Ciencias Exactas y Naturales, Universidad de Buenos Aires, Buenos Aires, Argentina (FCENCBPB); leaves are held under the numbers FCENCBPB 191–201 and infructescences under the numbers FCENCBPB 204 and 232.

Extant *Eucalyptus* flower buds and leaves were obtained from the LH Bailey Hortorium Herbarium (BH), Department of Plant Biology, Cornell University, Ithaca, NY, USA, and from the Herbario Parque Botánico Patagónico (PBP), MPEF. Leaves were cleared using standard protocols and were mounted in Cedarwood oil (Texas Fragrances, Leakey, Texas, USA), after a method suggested by Buechler [51].

### Phylogenetic Analyses

The hypothesized affinities of the fossils were tested within a phylogenetic context through analysis of morphological and molecular sequence datasets from extant taxa and morphological data from the fossils. The characteristics of the fossils were combined into a single terminal. We produced an updated morphological matrix by combining, modifying, and adding to characters from previously published morphological matrices [17], [24], [42], [52], [53]. In addition to analyzing this matrix alone, we fused our new morphological matrix with previously aligned molecular sequence data from four regions of DNA: 5S rDNA repeat, psbA-trnH spacer, trnL intron, and trnL 3′exon-trnF spacer [19]. Table S1 summarizes the way in which the taxa in the morphological matrix compiled for this study were combined with the taxa for which molecular data were obtained.

The molecular matrices used in this study consist of aligned molecular sequence datasets compiled and originally analyzed by Udovicic and Ladiges [19]. The aligned datasets were obtained directly from F. Udovicic, who provided them as .txt files in NEXUS format via e-mail. Five files were sent representing 1) 5S rDNA, 2) psbA and trnH spacer, 3) ITS-1 and ITS-2, 4) trnL intron, trnL 3′exon, and trnF spacer, 5) and a combined dataset. Recent analyses have revealed that some of the sequences in the ITS dataset are actually pseudogene paralogues of functional ITS sequences whereas some are functional genes [27], [54]. Thus, the ITS dataset was not used in the analyses in this paper, and a new combined dataset without this component was constructed. Aligned molecular sequence datasets were culled of uninformative and ambiguously aligned characters as described by Udovicic & Ladiges [19] using WinClada ver. 1.9 [55]. Resultant datasets were of the dimensions indicated in that publication, except that the psbA-trnH spacer dataset was two base pairs longer: 73 instead of 71 characters, including indels (the 73-character length conforms to the reported dimensions of the combined dataset in that paper [19], however). The culled 5S, psbA-trnH, and trnL-F datasets were combined into a single matrix of 160 characters and 17 taxa, including one outgroup taxon.

The morphological matrix includes 43 characters and 18 taxa, with one terminal representing the fossils (Appendices S1, S2). The matrix was constructed in WinClada ver. 1.9 [55] primarily using characters taken directly or modified from published morphological matrices by Ladiges & Humphries [52], Johnson & Briggs [42], Ladiges et al. [24], Hill & Johnson [17], and Wilson et al. [53]. Characters were selected to capture variation within the ingroup in this study. Autapomorphies and invariant characters were excluded from the morphological matrix. Taxa were often scored after previous published morphological matrices [9], [17], [24], [52], [53], although some additions and changes were made after reference to primary sources (See Appendix S1). Reference to the primary literature was made for all characters scored for *Allosyncarpia* and *Stockwellia* [15], [56]–[60]. Other terminals were scored at the generic, subgeneric, sectional, or species level, as applicable. Sometimes characters considered invariant at higher taxonomic levels were attributed to lower-level terminals where specific information for those terminals was lacking. The character “number of flowers per umbellaster” was scored at the species level using the exemplar species from the molecular sequence dataset (Table S1) for all terminals, since this character was highly variable.

The combined matrix is 203 characters and 18 taxa (Appendix S3). The Patagonian fossils are scored as missing for all molecular sequence and indel characters.

Analyses were performed including morphological data with extant taxa and the Patagonian fossils (Fig. S1), extant taxa with molecular sequence and indel characters (Fig. S2), and all characters and all taxa (Fig. 5). All analyses were launched from WinClada ver. 1.9 [55] and searches were performed in TNT ver. 1.1 for Windows [61], [62]. The same search parameters were used for each analysis under the parsimony criterion. A four-stage analysis of each data set was performed, using the parsimony ratchet, drift, and tree fusion, followed by swapping with TBR allowing up to 10,000 trees. This four-stage analysis was repeated 20 times with new random seeds to insure all tree space was adequately explored. These settings have been shown to be highly effective with data sets of this size range [63], [64]. All results were checked using the parsimony ratchet and 2000 iterations of branch-swapping with the program NONA ver. 2.0 for Windows [65] with exactly the same results as those found in TNT. Shortest trees were automatically submitted to WinClada. The “Hard collapse unsupported nodes” option was used to view tree topologies in the winClados interface in WinClada. Strict consensus trees were calculated using the “Nelsen” command, which also collapses nodes with only ambiguous character support. All tree statistics (length, CI, RI) were recorded as calculated in winClados before nodes were collapsed in most parsimonious trees.

### Figures and Imaging

The base map in Figure 1 was generated using the World Plate Caree template in ArcGIS 9 (©1999–2008 ESRI Incorporated) and modified in Adobe® Photoshop® CS4 Extended ver. 11.0 (©1990–2008 Adobe Systems Incorporated). Strict consensus trees generated in WinClada were saved in .emf format and opened in Adobe® Illustrator® CS4 ver. 14.0.0 (©1987–2008 Adobe Systems Incorporated), which was used to modify the trees for publication.

Overall views of fossil specimens (Figures 2A–B, D; 4A–D) were taken with a Nikon D500 Digital SLR Camera and images of extant flower buds and cleared leaves on herbarium sheets (Figures 2D–F; 3D) were taken with a Nikon D3X Digital SLR Camera. The extant leaf detail (Figure 2E) was taken with a Canon EOS 40D Digital SLR Camera. Magnified images (Figures 2C; 3A–D; 4E–F) were procured under a Nikon SMZ1000 dissecting microscope using a Nikon Digital Sight DS-Fi1 digital camera attached to a Nikon Digital Sight DS-L2 control unit. All multi-panel figures were composed using CorelDRAW® X5 version 15.1.0.588 (©2010 Corel Corporation). Brightness and/or contrast of all images were adjusted in CorelDRAW®. Temperature was adjusted in Figures 2A–C and 4B, E, F; tint was adjusted in Figure 4E. Gamma was adjusted in Figures 2B, D.

## Supporting Information

Figure S1 Strict consensus of 25 most parsimonious trees (length 85 steps, CI 0.62, RI 0.79) based on a phylogenetic analysis of 43 morphological characters for 17 extant taxa and the Patagonian fossils.
(TIFF)

Figure S2 Strict consensus of 2 most parsimonious trees (length 288 steps, CI 0.69, RI 0.79) based on a phylogenetic analysis of 160 molecular sequence and indel characters for 17 extant taxa.
(TIFF)

Table S1 Terminal names used in this study matched to terminal names used in the molecular analysis of Udovicic & Ladiges.
(DOC)

Appendix S1 Morphological character list. Characters and character states for the morphological matrix.
(DOC)

Appendix S2 Morphological dataset.
(TXT)

Appendix S3 Combined morphology and molecular dataset
(TXT)
