# Supplementary figures and images for: Oldest Known Eucalyptus Macrofossils Are from South America
Source: PLoS One. 2011 Jun 28;6(6):e21084. doi: 10.1371/journal.pone.0021084 (PMC3125177; doi:10.1371/journal.pone.0021084)

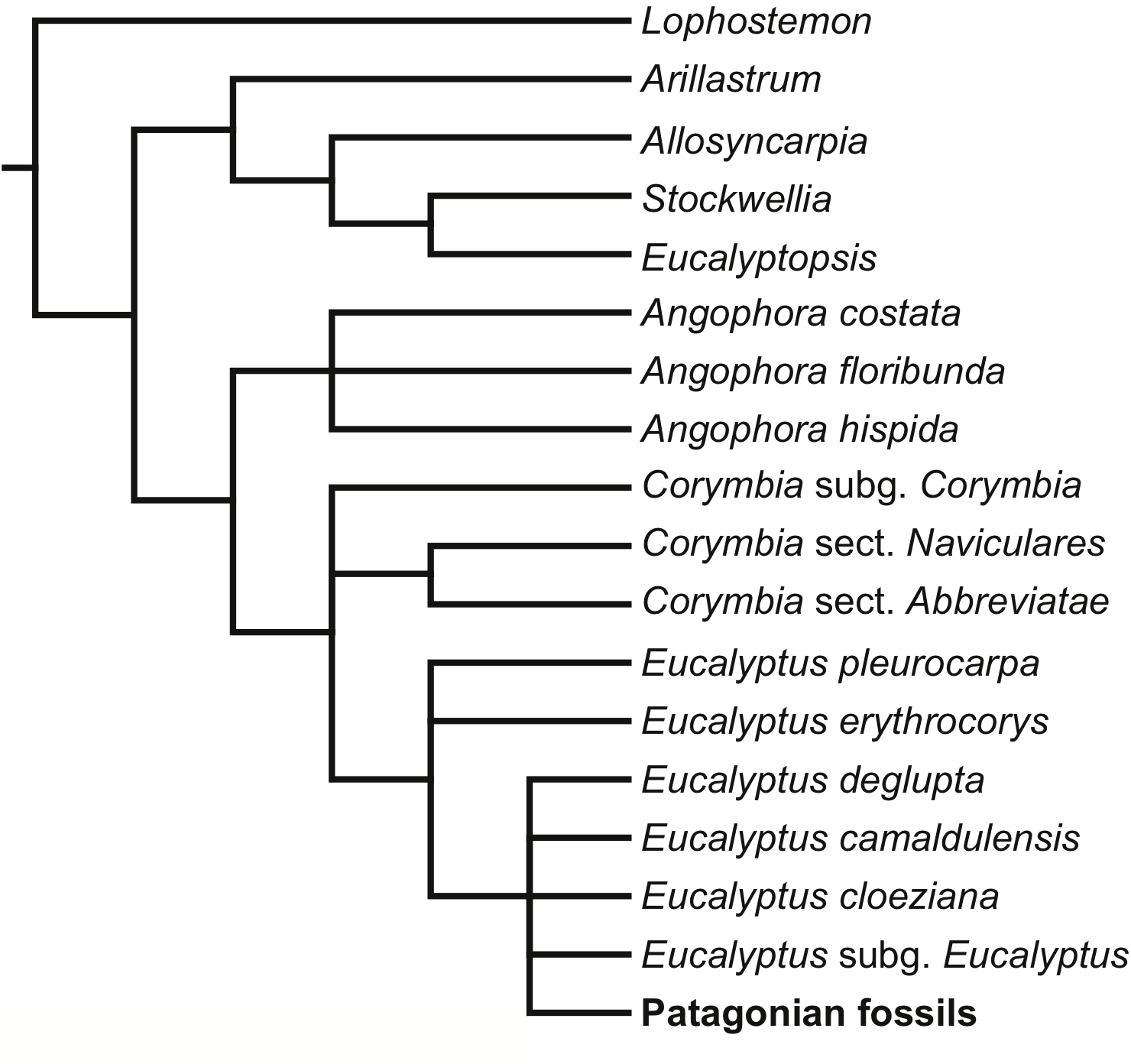

Supplement: Figure S1 — Strict consensus of 25 most parsimonious trees (length 85 steps, CI 0.62, RI 0.79) based on a phylogenetic analysis of 43 morphological characters for 17 extant taxa and the Patagonian fossils. (TIFF) [file pone.0021084.s001.tiff]

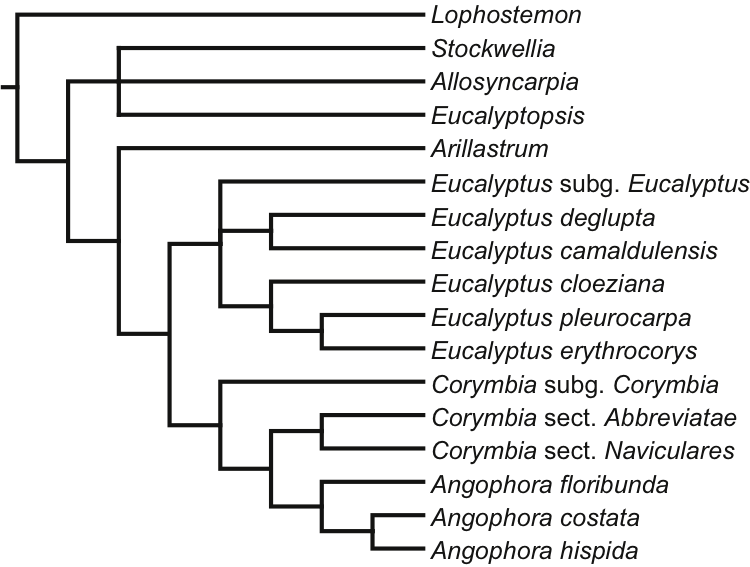

Supplement: Figure S2 — Strict consensus of 2 most parsimonious trees (length 288 steps, CI 0.69, RI 0.79) based on a phylogenetic analysis of 160 molecular sequence and indel characters for 17 extant taxa. (TIFF) [file pone.0021084.s002.tiff]
