# Supplementary material for: Oldest Known Eucalyptus Macrofossils Are from South America
Source: PLoS One. 2011 Jun 28;6(6):e21084. doi: 10.1371/journal.pone.0021084 (PMC3125177; doi:10.1371/journal.pone.0021084)
Supplement: Table S1 — Terminal names used in this study matched to terminal names used in the molecular analysis of Udovicic & Ladiges. (DOC) [file pone.0021084.s003.doc]

**Table S1. Terminal names used in this study matched to terminal names used in the molecular analysis of Udovicic & Ladiges** [19]**.**

| **This study** | **Udovicic & Ladiges** [19] | **Subgeneric classification (where applicable)** [9,66] |
| --- | --- | --- |
| *Lophostemon* | *Lophostemon* |  |
| *Allosyncarpia* | *Allosyncarpia* |  |
| *Arillastrum* | *Arillastrum* |  |
| *Eucalyptopsis* | *Eucalyptopsis* |  |
| *Stockwellia* | Myrtaceae sp. |  |
| *Angophora costata* | *A. costata* |  |
| *Angophora floribunda* | *A*. *floribunda* |  |
| *Angophora hispida* | *A*. *hispida* |  |
| *Corymbia* sect. *Abbreviatae* | *C*. *papuana* | subgenus *Blakella* |
| *Corymbia* sect. *Naviculares* | *C*. *eximia* | subgenus *Blakella* |
| *Corymbia* subg. *Corymbia* | *C. calophylla* | subgenus *Corymbia* |
| *Eucalyptus camaldulensis* | *E*. *camaldulensis* | subgenus *Symphyomyrtus* |
| *Eucalyptus cloeziana* | *E*. *clöeziana* | subgenus *Idiogenes* |
| *Eucalyptus deglupta* | *E. deglupta* | subgenus *Symphyomyrtus* |
| *Eucalyptus erythrocorys* | *E*. *erythrocorys* | subgenus *Eudesmia* |
| *Eucalyptus pleurocarpa* | *E*. *tetragona* | subgenus *Eudesmia* |
| *Eucalyptus* subg. *Eucalyptus* | *E*. *pilularis* | subgenus *Eucalyptus* |
| Patagonian fossils |  |  |

Terminal names in column one correspond to terminal names used in Appendices S2 and S3 and Figures 5, S1, and S2. The abbreviation “subg.” means subgenus, whereas “sect” stands for section. The circumscription of *Lophostemon* Schott follows Wilson & Waterhouse [81]. Subgenera of *Eucalyptus* follow the taxonomy of Brooker [66], except that *E*. *deglupta* is placed in subgenus *Symphyomyrtus* as suggested by the results of recent molecular sequence analyses [20,21]; subgenera and sections of *Corymbia* follow recent updates by Parra-O. et al. [9]. The name *E*. *tetragona* (R. Br.) F. Muell has been changed to *E*. *pleurocarpa* Schauer. The name *E*. *tetragona* is problematic, since the holotype of *E*. *tetragona* is thought to be an intergrade between the sister species *E*. *extrica* D. Nicolle and *E*. *pleurocarpa* [75,95]*.* Application of the name *E*. *pleurocarpa* to the plant providing the sequences in the Udovicic & Ladiges [19] datasets is the approach favored in other studies [27,54].
