# Supplementary material for: Oldest Known Eucalyptus Macrofossils Are from South America
Source: PLoS One. 2011 Jun 28;6(6):e21084. doi: 10.1371/journal.pone.0021084 (PMC3125177; doi:10.1371/journal.pone.0021084)
Supplement: Appendix S1 — Morphological character list. Characters and character states for the morphological matrix. (DOC) [file pone.0021084.s004.doc]

**Supporting Material**

**Appendix S1. Morphological character list**. References to all previously published matrices serving as sources for defining and/or scoring the characters as well as primary literature consulted in scoring are listed following each character. All characters are nonadditive. Character numbering corresponds to WinClada numbering; numbering for NEXUS files will start with 1 rather than 0.

*Stem Anatomy*

0. Vessel aggregation: 0) mostly solitary; 1) mostly grouped [17,42,53,56].

1. Paratracheal vertical parenchyma: 0) neither confluent nor banded (scanty); 1) either confluent or banded [17,42,53,56].

2. Crystalliferous strands in the vertical parenchyma: 0) absent; 1) present [42,53,56].

3. Stem, oil ducts: 0) absent; 1) present, large oil ducts in pith; 2) present, small oil ducts in pith and cortex [12,17,24,42,53,56,67,68].

*Leaves, Adult*

4. Adult leaves, phyllotaxy: 0) consistently opposite (opposite decussate); 1) at least partially alternate/spiral (opposite disjunct); 2) whorled [12,17,24,42,52,53,56,57,69-71].

5. Adult leaves, intramarginal vein: 0) absent; 1) present [12,17,42,53,56-58,72,73].

*Epidermal Structures, Juvenile, Intermediate, and/or Adult Leaves*

6. Angophoroid trichomes (multicellular, uniseriate, thin-walled, a2-type [74]): 0) absent; 1) present [17,42,52,53,74].

7. Emergent oil glands: 0) absent; 1) present, incipient condition (not elongated) or conspicuously elongated as bristle glands [11,17,24,42,52,53,74].

8. Oil glands, number of cap cells: 0) predominantly four per gland; 1) predominantly two per gland [17,24,74].

9. Oil gland cap cells: 0) without micropapillae; 1) with micropapillae [17,24,74].

10. Oil glands, radiating trichomes (r-type trichomes [11,74]): 0) absent; 1) present [9,11,17,24,52,71,74-76].

*Inflorescence*

11. Inflorescence phyllotaxy: 0) indefinite (disperse or flexible); 1) opposite [17,42,53,56,59].

12. Inflorescence branching: 0) panicles only; 1) panicles plus thrysoids, metabotryoids, etc.; 2) thrysoides, botryoides, etc., sometimes monads; 3) monads predominating [53,56,57].

13. Inflorescence bracts (recaulescence in inflorescence): 0) partially or completely fused to the inflorescence axis each subtends (recaulescence present); 1) free from the inflorescence axes (recaulescence absent) [42,53,56,57,59].

14. Number of flowers per umbellaster or equivalent: 0) 3; 1) 5-7; 2) >7. *New character*.

The umbellaster is the ultimate unit of the inflorescence in eucalypts and consists of flowers borne in an umbel-like arrangement [11,59,77]. The number of flowers per umbellaster can be fixed or variable within individual species. We have scored this character so that any state applying to a given terminal is scored as present. Due to the variability of this character within superspecific groups, it has been scored at the species level for all terminals using the exemplar species from the molecular sequence dataset (see Table S1). Multiple sources were consulted in scoring [10,13,17,56,57,69,71,78,79].

15. Flowers in umbellaster: 0) free; 1) fused. *New character*.

Connate flowers occur in *Allosyncarpia* and *Stockwellia* [15,56,57]. The hypanthia of flowers in *Eucalyptosis* can be fused or free [69,70].

16. Anthopodium [59] (e.g., pedicel): 0) present (anthopodium elongated); 1) absent, flowers sessile (anthopodium not elongated) [10,42,53,56,57].

17. *Arillastrum*-type trichomes (branched and septate trichomes) on inflorescence or floral parts (e.g., bracts, sepals, petals, hypanthium): 0) absent; 1) present [9,17,18,42,53,56,59].

18. Floral sclereids at anthesis: 0) absent (may develop in fruit); 1) present [26,52].

*General Perianth, Hypanthium, & Calyx*

19. Hypanthium abscission: 0) absent, hypanthium persistent; 1) circumscissile (abscission occurring with hypanthium and attached perianth lobes lost as a unit). *New character*, based on discussion in Bohte & Drinnan [15].

20. Perianth mery: 0) 5(-6); 1) not fixed, 4 and 5; 2) fixed, 4 [13,15,17,42,53,56,57,71,78].

21. Calyx: 0) sepals free; 1) sepals connate, forming an operculum [10,15-17,24,52,57,80].

22. Calyx (free or opercular): 0) persistent, often to fruiting stage; 1) shed at anthesis with corolla; 2) caducous [10-12,15-17,24,52,71,78,80-83].

*Corolla & Androecium*

23. Corolla morphology: 0) well-developed, petals free or corolla opercular; 1) petals highly reduced, corolla rudimentary. *New character*, based on descriptions of the corollas of *Eucalyptopsis* and *Stockwellia* in Bohte & Drinnan [15].

24. Corolla: 0) petals free (but may adhere to calyx); 1) petals coherent (margins discernable); 2) petals connate (margins usually not discernable) [9,10,15,17,24,25,42,52].

25. Corolla (free or opercular): 0) persistent; 1) lost at anthesis [10,13,15,78]. *New character*.

26. Petals with keel-limb structure (e.g., staminophore or homologue): 0) absent; 1) present (represented as adaxial petaloid limbs not bearing stamens or as a single circumfloral buttress or series of antepetalous buttresses centripetal to the corolline whorl and bearing stamens) [15,16,24,25,80,82,84,85].

27. Petal limb morphology at anthesis: 0) petal limbs (stamens not epipetalous); 1) staminophore (buttress or series of buttresses centripetal to the corolla whorl bearing stamens; stamens epipetalous) [17,25].

28. Petal limb/staminophore development: 0) beginning as separate buttresses and either remaining distinct or becoming continuous; 1) continuous circumfloral ring throughout development [24,25,52].

*Gynoecium*

29. Carpel number: 0) 1-2; 1) 3; 2) 4; 3) 5; 4) 6 [10,12,13,17,42,53,56,57,69-73,78,81].

30. Style tip engaged in pit in corolla before anthesis: 0) absent; 1) present [9,15-17,25,26,72,80,82,84,85].

31. Stigma papillae: 0) long (greater than 60 μm); 1) short (less than 60 μm) [17,24,71,86].

*Ovule, Seed, & Embryo*

32. Ovule arrangement: 0) not in distinct rows (proximal-lateral-distal placenta expansion); 1) in two to ten vertical rows (proximal-lateral placenta expansion) [17,24,42,52,60,71,87]. This character is based on the arrangement of the developed ovules on the placenta as scored in previous matrices [24,52] and supplemented by observations of placental development by Bohte & Drinnan [60].

33. Ovules: 0) anatropous; 1) hemitropous; 2) campylotropous [11,17,24,42,52,53,56,57,69,77,81].

34. Fruit valves: 0) enclosed; 1) level to exserted [10,12,17,56,57,70,71,73,79]. *New character*.

35. Disc in fruit: 0) descending; 1) level; 2) ascending [10,12,15,71]. *New character*.

36. Number of seeds in fruit : 0) greater than 1 per locule; 1) 1 per locule ; 2) less than 1 per locule [10,13,42,53,56,57,60,69,78,88].

37. Seeds at maturity: 0) bitegmic with the inner clearly developed and suberized; 1) unitegmic or bitegmic with the inner unsuberized and often partly resorbed [17,24,52,78,89-92].

38. Well-organized crystalliferous layer in the seed testa (outer integument with crystalliferous inner epidermis): 0) absent; 1) present [17,24,42,52,53].

39. Outer integument of the ovule/seed: 0) 2 cells thick; 1) 4 cells thick; 2) 6-8 cells thick [17,24,52,93].

40. Mature seed, cracking of the seed coat: 0) absent; 1) present [9,17,75,83].

41. Mature seed, raphe: 0) absent; 1) present [17,90-92].

42. Cotyledons: 0) entire; 1) emarginated [17,24,52,56,94].

**References**

67.     Welch MB (1921) The occurrence of oil ducts in certain eucalypts and angophoras. Proc Linn Soc N S W 46: 475-486, plates 42-46.

68.     Carr SGM, Carr DJ (1969) Oil glands and ducts in *Eucalyptus* L'Hérit. I. The phloem and the pith. Aust J Bot 17: 471-513.

69.     White CT (1951) Some noteworthy Myrtaceae from the Moluccas, New Guinea, and the Solomon Islands. J Arnold Arboretum 32: 139-149.

70.     Craven LA (1990) One new species each in *Acema* and *Eucalyptopsis* and a new name in *Lindsayomyrtus* (all Myrtaceae). Aus Syst Bot 3: 727-732.

71.     Hill KD, Johnson LAS (1998) Systematic studies in the eucalypts. 8. A review of the Eudesmioid eucalypts, *Eucalyptus* subgenus *Eudesmia* (Myrtaceae). Telopea 7: 375-414.

72.     Brooker MIH, Bean AR (1991) A revision of the yellow bloodwoods (Myrtaceae: *Eucalyptus* ser. *Naviculares* Maiden). Austrobaileya 3: 409-437.

73.     Conn BJ, Damas KQ. (2006+) Guide to Trees of Papua New Guinea. Available: http://www.pngplants.org/PNGtrees. Accessed 2010 Nov 10.

74.     Ladiges PY (1984) A comparative study of trichomes in *Angophora* Cav. and *Eucalyptus* L'Hérit.—a question of homology. Aust J Bot 32: 561-574.

75.     Gibbs AK, Udovicic F, Drinnan AN, Ladiges PY (2009) Phylogeny and classification of *Eucalyptus* subgenus *Eudesmia* (Myrtaceae) based on nuclear ribosomal DNA, chloroplast DNA and morphology. Aus Syst Bot 22: 158-179.

76.     Carr DJ, Carr SGM (1980) The *Lehmannianae*: a natural group of western Australian eucalypts. Aust J Bot 28: 523-550.

77.     Johnson LAS (1976) Problems of species and genera in *Eucalyptus* (*Myrtaceae*). Pl Syst Evol 125: 155-167.

78.     Dawson JW (1970) Pacific capsular Myrtaceae I. Reproductive morphology of *Arillastrum gummiferum* Panch. ex Baillon (New Caledonia). Blumea 18: 431-440.

79.     Wagner WL, Herbst DR, Sohmer SH (1999) Manual of the flowering plants of Hawai'i. Honolulu: University of Hawai'i Press. 988 p.

80.     Drinnan AN, Ladiges PY (1989) Operculum development in *Eucalyptus clöeziana* and *Eucalyptus* informal subg. *Monocalyptus* (*Myrtaceae*). Pl Syst Evol 166: 183-196.

81.     Wilson PG, Waterhouse JT (1982) A review of the genus *Tristania* R. Br. (Myrtaceae): a heterogenous assemblage of five genera. Aust J Bot 30: 413-446.

82.     Drinnan AN, Ladiges PY (1989) Operculum development in the *Eudesmieae* B eucalypts and *Eucalyptus caesia* (*Myrtaceae*). Pl Syst Evol 165: 227-237.

83.     Boland DJ, Brooker MIH, Turnbull JW (1980) *Eucalyptus* Seed. Camberra, Australia: CSIRO. 191 p.

84.     Drinnan AN, Ladiges PY (1989) Corolla and androecium development in some *Eudesmia* eucalypts (*Myrtaceae*). Pl Syst Evol 165: 239-254.

85.     Orlovich DA, Drinnan AN, Ladiges PY (1996) Floral development in the *Metrosideros* group (Myrtaceae) with special emphasis on the androecium. Telopea 6: 689-719.

86.     Boland DJ, Sedgley M (1986) Stigma and style morphology in relation to taxonomy and breeding systems in *Eucalyptus* and *Angophora* (Myrtaceae). Aust J Bot 34: 569-584.

87.     Carr SGM, Carr DJ (1962) Convergence and progression in *Eucalyptus* and *Symphyomyrtus*. Nature 196: 969-972.

88.     Leach GJ (1986) A revision of the genus *Angophora* (Myrtaceae). Telopea 2: 749-779.

89.     Prakash N (1969) A contribution to the life history of *Angophora floribunda* (Sm.) Sweet (Myrtaceae). Aust J Bot 17: 457-469.

90.     Gauba E, Pryor LD (1959) Seed coat anatomy and taxonomy in *Eucalyptus*. II. Proc Linn Soc N S W 84: 278-291, plates 11-13.

91.     Gauba E, Pryor LD (1961) Seed coat anatomy and taxonomy in *Eucalyptus*. III. Proc Linn Soc N S W 86: 96-111, plate 3.

92.     Gauba E, Pryor LD (1958) Seed coat anatomy and taxonomy in *Eucalyptus*. I. Proc Linn Soc N S W 83: 20-32, plate 1.

93.     Mauritzon J (1939) Contributions to the embryology of the orders Rosales and Myrtales. Kongl Fysiogr Saellsk Lund Handl NF 35: 1-121.

94.     Hyland BPM, Whiffin T (1993) Australian tropical rain forest trees, an interactive identification system volume 1. East Melbourne, Victoria, Australia: CSIRO. 564 p.

95.     Nicolle D (2000) Three new taxa of *Eucalyptus* subgenus *Eudesmia* (Myrtaceae) from Queensland and Western Australia. Nuytsia 13: 317-329.
